# Post percutaneous coronary interventional outcomes on proximal vs non-proximal lesions of the left and right coronary arteries: A systematic review and meta-analysis

**DOI:** 10.1097/MD.0000000000016905

**Published:** 2019-08-16

**Authors:** Bing Tang, Hua Yang

**Affiliations:** Department of Cardiology, Jingzhou Central Hospital, The Second Clinical Medical College, Yangtze University, Jingzhou, Hubei, PR China.

**Keywords:** coronary artery disease, left proximal coronary artery lesion, major adverse cardiac events, mortality, percutaneous coronary intervention, right proximal coronary lesion

## Abstract

**Background:**

The prognosis of patients with coronary artery disease is mainly related to the extent of myocardium at risk. Proximal coronary arteries, especially the proximal left anterior descending coronary artery (LAD), supply a large part of the myocardium. In this analysis, we aimed to systematically compare the post percutaneous coronary interventional (PCI) outcomes observed with proximal vs non-proximal lesions of the left and right coronary arteries.

**Methods:**

MEDLARS Online, Excerpta Medica database, www.ClinicalTrials.gov, and the Cochrane databases were searched for relevant studies comparing the post PCI outcomes reported on proximal vs non-proximal lesions of the coronary arteries. RevMan software version 5.3 was used to analyze the data to generate respective results. Odds ratios (OR) and 95% confidence intervals (CI) were derived to represent the results appropriately.

**Results:**

Six studies with a total number of 11,109 participants who were enrolled between 1990 and 2015 were included in this analysis. The current results showed major adverse cardiac events (MACEs) (OR: 1.28, 95% CI: 1.14–1.45; *P* = .0001) and mortality (OR: 1.70, 95% CI: 1.43–2.03; *P* = .00001) to be significantly higher with proximal compared to non-proximal coronary lesions irrespective of the follow-up time periods. However, re-infarction (OR: 1.05, 95% CI: 0.80–1.38; *P* = .71), repeated revascularization (OR: 1.08, 95% CI: 0.92–1.27; *P* = .35) and stent thrombosis (OR: 0.59, 95% CI: 0.27–1.31; *P* = .20) were not significantly different.

When patients specifically with LAD lesions were compared with associated non-proximal lesions, mortality was still significantly higher with proximal lesions (OR: 2.26, 95% CI: 1.52–3.36; *P* = .0001). However, when patients with right proximal coronary artery lesions were compared with the corresponding non-proximal lesions, no significant difference was observed in mortality.

**Conclusion:**

In-hospital and long-term MACEs and mortality were significantly higher in patients with proximal compared to non-proximal coronary lesions following PCI. In addition, mortality was significantly higher in patients with proximal LAD lesions whereas no significant difference was observed in patients with right proximal coronary artery lesions. Larger trials should further confirm these hypotheses.

## 1 Introduction

Coronary artery disease (CAD) and percutaneous coronary intervention (PCI) have been 1 among the most common diseases and treatment option, respectively in cardiac centers.^[1,2]^ Estimates from the World Health Organization predict CAD to be the main cause of mortality worldwide with values which exceeded 9 million deaths in 2016.^[1]^ Moreover, by accounting for almost 31% of all mortality throughout the globe, CAD is considered as the leading cause of death and is expected to maintain this position until 2030.^[3]^ Therefore, the hospital burden for CAD and PCI has increased recently. Data of the national French Prospective Payment System database from the years 2009 to 2014, consisting of French patients living in Metropolitan showed a total number of 678,021 patients with CAD over this 6-year period, representing 900,121 hospital stay, with 215,224 patients with myocardial infarction, or underwent revascularization with PCI.^[2]^

While most of us are aware of the severity and consequences of an acute coronary syndrome (ACS), it is less known that the prognosis of patients with CAD is also mainly related to the extent of myocardium which has been damaged.^[4]^ Proximal coronary arteries, especially the proximal left anterior descending coronary artery (LAD) normally supply a large part of the myocardium when compared to the non-proximal arteries. To be more clear on this matter, occlusion of the proximal part of LAD might result in ischemia in a significant myocardial territory, and is associated with a poor prognosis.^[5]^ However, the outcomes associated with proximal vs non-proximal coronary lesions following PCI have seldom been systematically assessed.

In a cross sectional study, the authors stated that being aware of the fact that proximal LAD lesions are associated with worse prognosis, they observed similar outcomes in PCI on proximal LAD, vs proximal left circumflex artery/right coronary artery (RCA) and non-proximal LAD.^[6]^ In a single centered study whereby 1468 patients were analyzed, infarcts related to proximal LAD were associated with worse prognosis when compared to distal LAD or non-LAD related infarcts.^[7]^ In this analysis, we aimed to systematically compare the post PCI outcomes observed with proximal vs non-proximal lesions of the left and right coronary arteries.

## 2 Methods

### 2.1 Databases to retrieve relevant studies

The Medical Literature Analysis and Retrieval System Online (MEDLINE), also known as MEDLARS Online, Excerpta Medica database (EMBASE), Resources from the United States National Library of Medicine (www.ClinicalTrials.gov: http://www.clinicaltrials.gov) and the Cochrane databases were the search databases used to retrieve relevant studies comparing the post PCI outcomes reported on proximal vs non-proximal lesions of the coronary arteries.

### 2.2 Terms used to retrieve studies

The following terms or phrases were used to retrieve English publications from the above mentioned search databases: “proximal coronary lesions and percutaneous coronary intervention”, “proximal lesions and coronary angioplasty”, “proximal and non-proximal lesions and percutaneous coronary intervention”, “proximal and non-proximal lesions and PCI”, “proximal versus distal lesions and percutaneous coronary intervention”, “left proximal anterior descending lesions and percutaneous coronary intervention”, “left proximal main coronary lesions and percutaneous coronary intervention”, “right proximal coronary lesions and percutaneous coronary intervention”, “proximal coronary lesions, outcomes and percutaneous coronary intervention”.

These search terms were used to retrieve articles from each of the electronic databases. Manual search was not necessary.

Retrieval of publications was dependent upon the PRISMA guideline.^[8]^

### 2.3 Two main criteria which were considered for the inclusion of studies

Studies were included if:

(1) They were randomized trials or observational (prospective, retrospective, cross sectional) studies comparing post percutaneous coronary interventional outcomes in patients with proximal vs non-proximal coronary lesions (including left proximal anterior descending coronary artery, left proximal circumflex artery, and/or right proximal coronary artery lesions;
(2) They had an in-hospital, short-term or longer follow-up time period.

### 2.4 Criteria which were considered for exclusion of studies

Studies were excluded if:

(1) They were review of literature/case studies/meta-analysis/letters to editors;
(2) They did not report post percutaneous coronary interventional outcomes;
(3) They did not compare proximal vs non-proximal coronary lesions;
(4) They were duplicated studies or they were repeated in several different search databases.

### 2.5 Types of participants, coronary lesions involved, outcomes reported, and the follow-up time periods

Patients with CAD including mainly STEMI undergoing revascularization by PCI were included in this analysis. Most of the lesions involved were from the LAD arteries. However, patients having lesions on the left proximal circumflex artery and the right proximal coronary artery were also included as shown in Table 1.

**Table 1:**
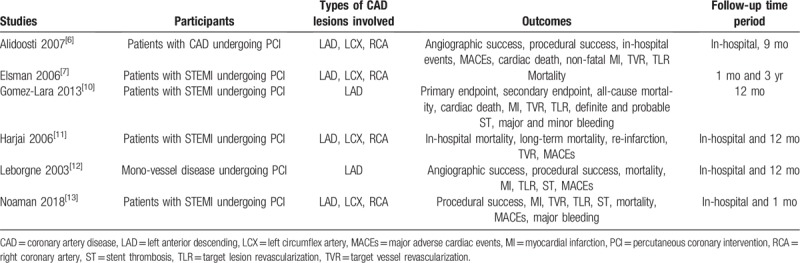
Participants, outcomes, and follow-up time periods.

The main post percutaneous coronary interventional outcomes that were reported included:

(1) Major adverse cardiac events (MACEs);
(2) Mortality (all-cause mortality including cardiac death);
(3) Re-infarction;
(4) Repeated revascularization;
(5) Stent thrombosis.

An in-hospital follow-up as well as a longer follow-up time period (9 months to 3 years) were considered as listed in Table 1.

### 2.6 Data extraction and quality assessment

Two experts independently extracted all the required data including the total number of participants assigned to the respective groups, the corresponding post percutaneous coronary interventional outcomes which were reported, the time period of follow-up, the types of coronary arteries affected, the types of participants who underwent PCI, the baseline features (age, gender, cardiovascular risk factors), the time period of patients’ enrollment, the total number of events, and other methodological features of the relevant studies. Any disagreement which occurred was discussed and then resolved by consensus.

The 2 experts also assessed the methodological features of the relevant studies with reference to the recommended criteria proposed by the Cochrane collaboration.^[9]^

### 2.7 Statistical methods used for data analysis

RevMan software version 5.3 was used to analyze the data to generate respective results. Odds ratios (OR) and 95% confidence intervals (CI) were derived to represent the results appropriately. Heterogeneity was assessed by the common *Q* statistic test whereby a *P* value less than .05 following subgroup analysis was considered as a statistically significant result. In addition, heterogeneity was also assessed by the *I* ^2^ statistic test whereby a low heterogeneity was represented by a low *I* ^2^ value. A fixed statistical model was used throughout the analysis.

Sensitivity analysis was also carried out based on an exclusion method whereby the concerned studies were excluded one by one and a new analysis was carried out each time and compared for any significant difference from the main result. In addition, funnel plots which were derived from the same RevMan software was used to visually assess for publication bias.

The following terms which appeared in the figures were defined as followed:

Odds ratios (OR): is defined as a measure of the association between an exposure and an outcome. OR represents the odds that a specific outcome or endpoint will occur when given a particular exposure in comparison to the same outcome occurring in the absence of that exposure.

CI: is defined as an interval estimate consisting of the true value of an unknown population parameter.

Standard error (SE): this is normally the standard error for treatment effect in this analysis.

### 2.8 Ethical compliances

No ethical approval/No board review approval was required for this meta-analysis, since data were not obtained through experiments on animals or human being carried out by any of the authors. Data were extracted from previously published original studies. Permission and ethical approval were granted to the authors of the original studies. References have been provided for the original studies which were used in this review, and original data can directly be accessed without any restriction.

## 3 Results

### 3.1 Outcomes following the search process

Search carried out by the 2 independent experts from online databases resulted in a total number of 98 publications. After carefully consulting each other by discussing abstracts and titles, 70 articles were eliminated since they were not based on this specific idea. Twenty-eight (28) full-text articles were assessed for possible eligibility.

Further studies were eliminated because they were: case studies (2), and repeated studies (20).

**Figure 1:**
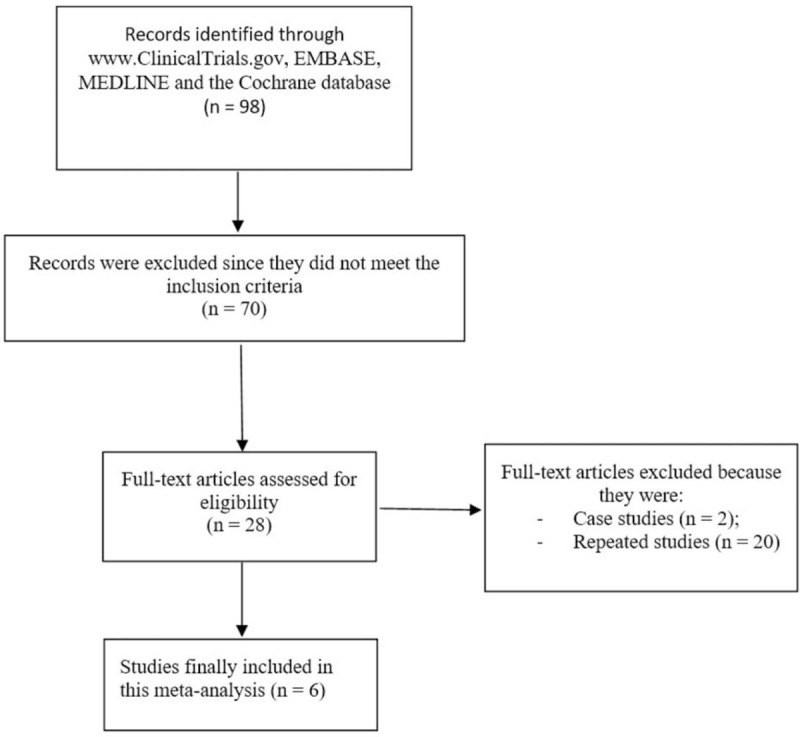
Flow diagram showing study selection.

Finally only 6 studies^[6,7,10,13]^ were selected for this meta-analysis as shown in Figure 1.

### 3.2 Main features of the involved studies

Six studies with a total number of 11,109 participants were enrolled in this analysis. Four thousand seven hundred ninety-two (4792) participants were assigned to the proximal lesion group whereas 6317 participants were assigned to the non-proximal lesion group. Participants were enrolled from the year 1990 to 2015. The detailed number of participants extracted from each study has been listed in Table 2.

**Table 2:**
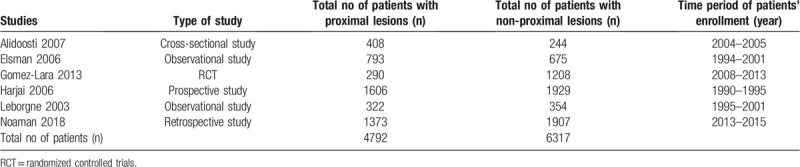
Main features of the studies.

Table 2 also included the type of study (randomized trial, observational study, cross sectional study, prospective and retrospective studies).

### 3.3 Baseline features of the involved participants

The mean age of the participants (55.4–62.0 years), the percentage of males (66.4–83.8%), and the percentage of patients with several cardiovascular risk factors including hypertension (21.0–60.5%), diabetes mellitus (8.00–24.0%), dyslipidemia (14.0–65.6%), and current smoker (20.7–73.0%) have been listed among the baseline features in Table 3.

**Table 3:**
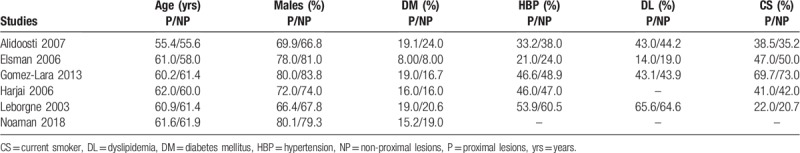
Baseline characteristics of the respective participants which were included in this analysis.

Other characteristics, such as the percentage of patients with multi-vessel diseases, reference vessel diameter, direct stenting technique, stent length, and stent diameter have been listed in Table 4 if they were reported.

**Table 4:**
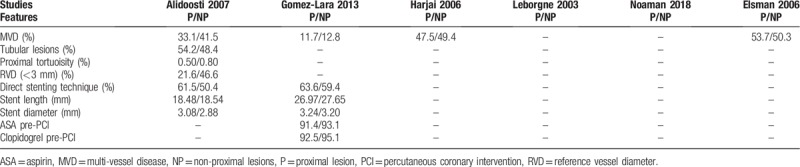
Other characteristics of the lesions involved among the participants.

### 3.4 Post percutaneous coronary interventional outcomes on proximal vs non-proximal lesions

**Figure 2:**
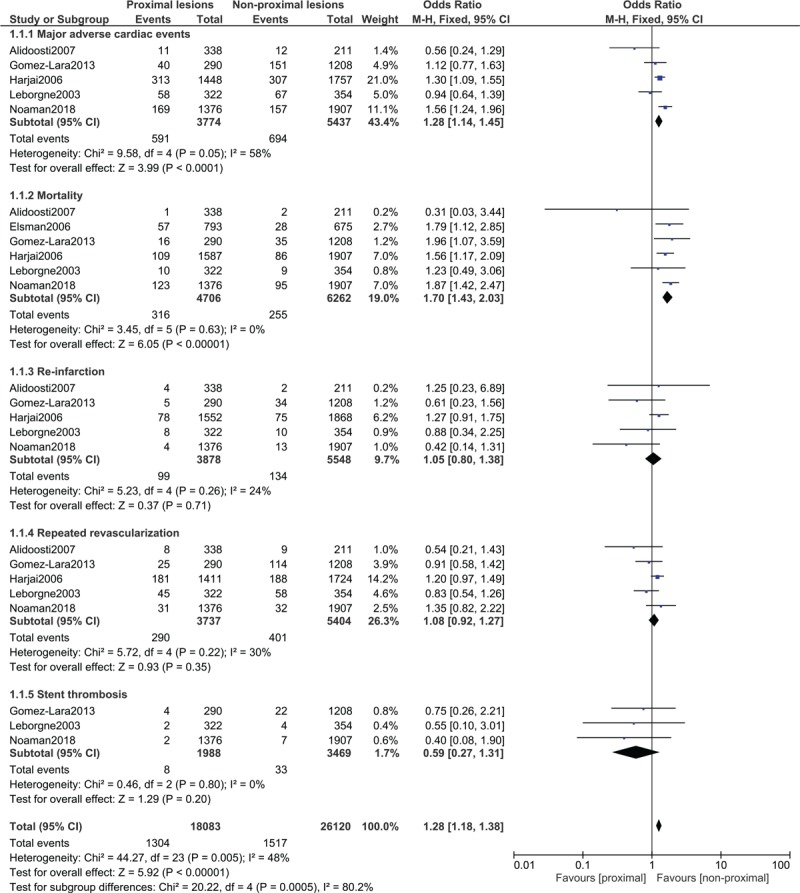
Post percutaneous coronary interventional outcomes on proximal vs non-proximal lesions of the left and right coronary arteries. CI = confidence intervals, M-H = Mantel Haenszel, Z = overall effect.

When post percutaneous coronary interventional outcomes were compared in patients with proximal vs non-proximal coronary lesions, MACEs (OR: 1.28, 95% CI: 1.14–1.45; *P* = .0001) and mortality (OR: 1.70, 95% CI: 1.43–2.03; *P* = .00001) were significantly higher with proximal coronary lesions as shown in Figure 2. However, re-infarction (OR: 1.05, 95% CI: 0.80–1.38; *P* = .71), repeated revascularization (OR: 1.08, 95% CI: 0.92–1.27; *P* = .35) and stent thrombosis (OR: 0.59, 95% CI: 0.27–1.31; *P* = .20) were not significantly different (Fig. 2).

**Figure 3:**
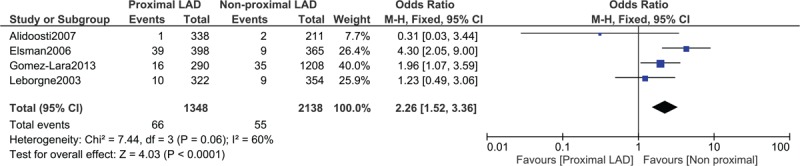
Post percutaneous coronary interventional outcomes on proximal vs non-proximal lesions of the left anterior descending artery. CI = confidence intervals, LAD = left anterior descending artery, M-H = Mantel Haenszel, Z = overall effect.

**Figure 4:**
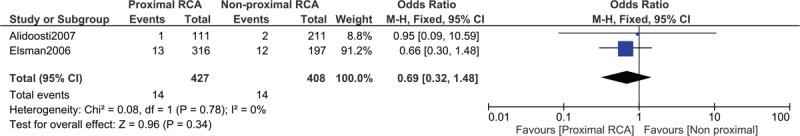
Post percutaneous coronary interventional outcomes on proximal vs non-proximal lesions of the right coronary artery. CI = confidence intervals, M-H = Mantel Haenszel, RCA = right coronary artery, Z = overall effect.

The outcomes were further classified according to lesions sites. When patients with left proximal anterior descending artery were compared with associated non-proximal lesions, mortality was still significantly higher with proximal lesions (OR: 2.26, 95% CI: 1.52–3.36; *P* = .0001) as shown in Figure 3. However, when patients with right proximal coronary artery lesions were compared with the corresponding non-proximal lesions, no significant difference was observed in mortality (OR: 0.69, 95% CI: 0.32–1.48; *P* = .34) as shown in Figure 4.

Outcomes were also classified according to follow-up time periods.

**Figure 5:**
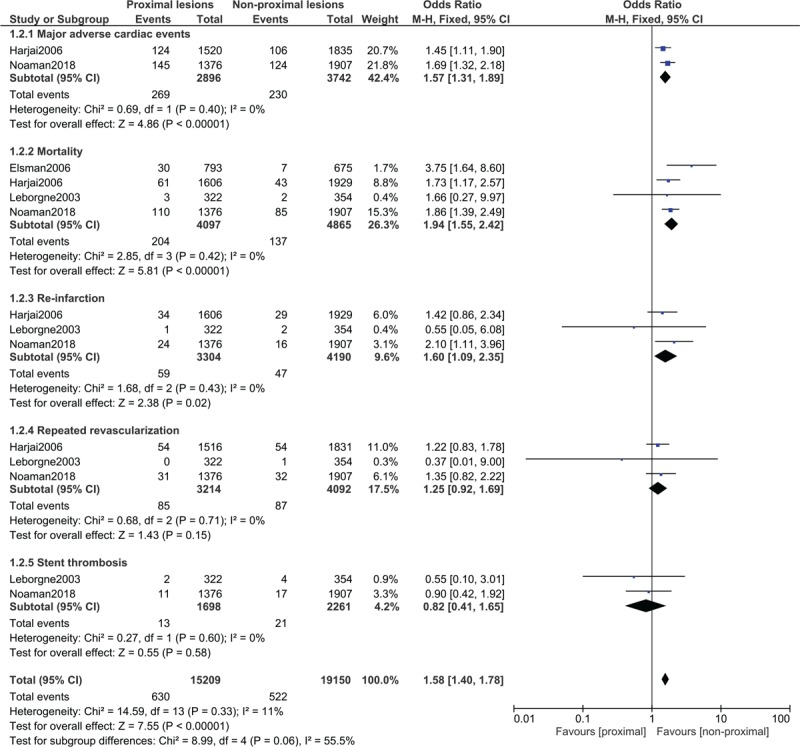
Post percutaneous coronary interventional outcomes on proximal vs non-proximal lesions of the left and right coronary arteries during the in-hospital follow-up time period. CI = confidence intervals, M-H = Mantel Haenszel, Z = overall effect.

During the in-hospital follow-up, MACEs (OR: 1.57, 95% CI: 1.31–1.89; *P* = .00001), mortality (OR: 1.94, 95% CI: 1.55–2.42; *P* = .00001) and re-infarction chances (OR: 1.60, 95% CI: 1.09–2.35; *P* = .02) were significantly higher in the proximal lesions group as shown in Figure 5. However, repeated revascularization (OR: 1.25, 95% CI: 0.92–1.69; *P* = .15) and stent thrombosis (OR: 0.82, 95% CI: 0.41–1.65; *P* = .58) were not significantly different (Fig. 5).

**Figure 6:**
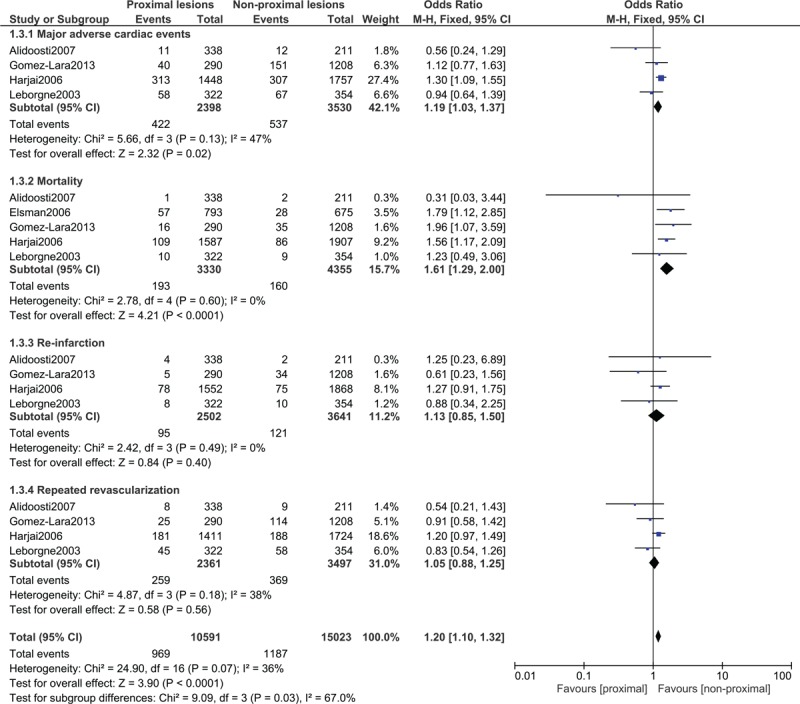
Post percutaneous coronary interventional outcomes on proximal vs non-proximal lesions of the left and right coronary arteries during the long-term follow-up time period. CI = confidence intervals, M-H = Mantel Haenszel, Z = overall effect.

During the longer follow-up time period, MACEs (OR: 1.19, 95% CI: 1.03–1.37; *P* = .02) and mortality (OR: 1.61, 95% CI: 1.29–2.00; *P* = .0001) were still significantly higher in patients with proximal coronary lesions as shown in Figure 6. However, re-infarction (OR: 1.13, 95% CI: 0.85–1.50; *P* = .40) and repeated revascularization (OR: 1.05, 95% CI: 0.88–1.25; *P* = .56) were not significantly different (Fig. 6).

A summarized version of the results has been given in Table 5.

**Table 5:**
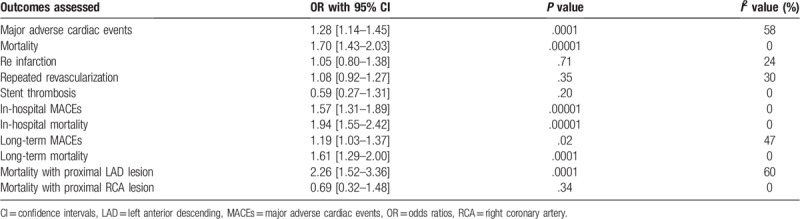
Summarized version of the results.

### 3.5 Sensitivity analysis and publication bias

**Figure 7:**
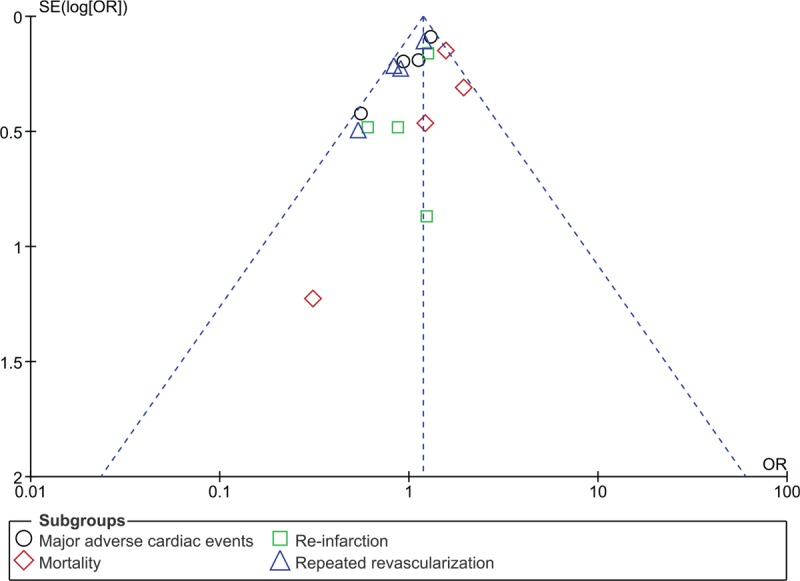
Funnel plot representing publication bias (A). OR = odds ratios, SE = standard error.

**Figure 8:**
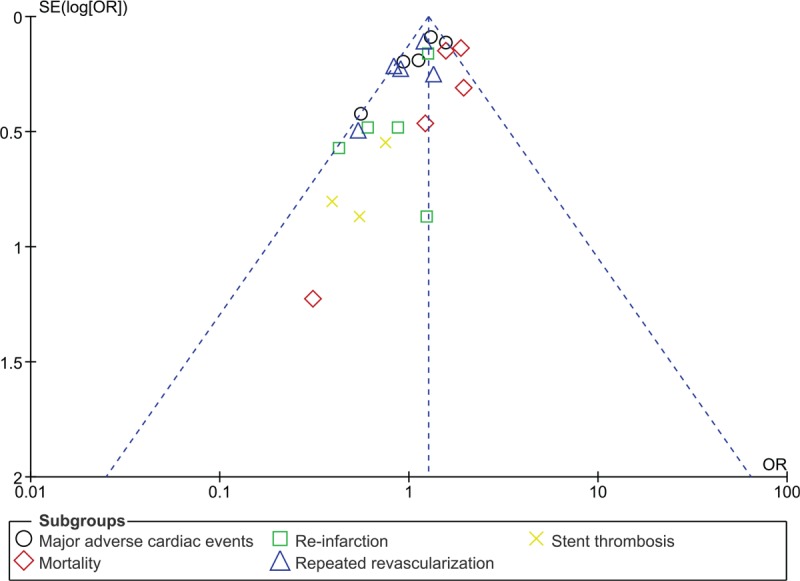
Funnel plot representing publication bias (B). OR = odds ratios, SE = standard error.

Consistent results were obtained when each study was excluded by turn, and a new analysis was carried out for sensitivity analyses. In addition, due to the smaller volume of studies, publication bias was visually observed from the funnel plots which were derived from the RevMan software: with funnel plots showing low evidence of publication bias as demonstrated in Figures 7 and 8.

## 4 Discussion

The location of a coronary lesion is also an integral part in order to predict prognosis following PCI. The current results showed proximal coronary lesions to be associated with significantly higher MACEs and mortality following PCI in comparison to the non-proximal coronary lesions. In-hospital and long-term follow-up time periods showed the same results. When mortality was compared, a higher rate was associated with lesions of the LAD. However, lesions of the right proximal coronary artery were not associated with significantly higher mortality.

Similarly, another analysis involving patients with STEMI undergoing PCI showed participants with proximal LAD lesions to have significantly worse prognosis and were associated with a significantly higher rate of 3-year mortality following the interventional procedure.^[7]^

Even if we might not rely on the results obtained for proximal RCA due to the very limited number of participants involved in the analysis, pooled clinical data from the Primary Angioplasty for Myocardial Infarction (PAMI) generated results supporting this current analysis for the fact that post angioplasty cardiovascular outcomes associated with lesions from the proximal RCA were similar compared to their non-proximal counterparts.^[14]^

Other studies have also shown significant lumen obstruction in the segments proximal to myocardial bridge to also have higher chances of re-stenosis and the occurrence of an increased amount of MACEs following PCI with drug eluting stents.^[15]^

Nevertheless, even if it is known that lesions from the left proximal anterior descending artery are associated with worse outcomes following PCI, another interesting cross-sectional study showed similar long-term post percutaneous coronary interventional outcomes associated with lesions within the proximal left anterior descending, proximal left circumflex coronary artery, RCA and non-proximal ones.^[6]^ However, it should be noted that their comparison was among all the different vessels whereas in this analysis, all the different coronary artery lesions were being compared with the non-proximal ones which might have turned out to be different but reasonable.

First, limitations which were reported included but was not restricted to a shortage of participants. Second, when comparing the long-term outcomes, different studies had different long-term follow up time periods ranging from 9 months to 3 years. This could have had an impact on the results which were generated. Another limitation could be the fact that the total number of participants which were involved in assessing the right proximal coronary artery lesions were less compared to the other subgroups. Hence, it would not be good to rely on this particular result for this specific subgroup until further major studies have confirmed this hypothesis. This analysis consisted mainly of patients with STEMI and the results might not apply to other types of ACS or patients with stable CAD and should only be proven in future studies.

## 5 Conclusion

In-hospital and long-term MACEs and mortality were significantly higher in patients with proximal lesions as compared to non-proximal lesions following PCI. In addition, mortality was significantly higher in patients with left proximal anterior descending lesions whereas no significant difference was observed in patients with right proximal coronary artery lesions. Larger trials should further confirm these hypotheses.

## Author contributions

**Conceptualization:** Bing Tang, Hua Yang.

**Data curation:** Bing Tang, Hua Yang.

**Formal analysis:** Bing Tang, Hua Yang.

**Funding acquisition:** Bing Tang, Hua Yang.

**Investigation:** Bing Tang, Hua Yang.

**Methodology:** Bing Tang, Hua Yang.

**Project administration:** Bing Tang, Hua Yang.

**Resources:** Bing Tang, Hua Yang.

**Software:** Bing Tang, Hua Yang.

**Supervision:** Bing Tang, Hua Yang.

**Validation:** Bing Tang, Hua Yang.

**Visualization:** Bing Tang, Hua Yang.

**Writing – original draft:** Bing Tang, Hua Yang.

**Writing – review & editing:** Bing Tang, Hua Yang.
